# Reclassification of endometrial cancer and identification of key genes based on neural-related genes

**DOI:** 10.3389/fonc.2022.951437

**Published:** 2022-09-23

**Authors:** Fan Chen, Tiansheng Qin, Yigan Zhang, Linzhen Wei, Yamei Dang, Peixia Liu, Weilin Jin

**Affiliations:** ^1^ The First Clinical Medical College of Gansu University of Chinese Medicine (Gansu Provincial Hospital), Lanzhou, China; ^2^ Key Laboratory of RNA Biology, Institute of Biophysics, Chinese Academy of Sciences, Beijing, China; ^3^ Department of Obstetrics and Gynecology, Yuzhong County Hospital of Traditional Chinese Medicine, Lanzhou, China; ^4^ Institute of Cancer Neuroscience, Medical Frontier Innovation Research Center, The First Hospital of Lanzhou University, The First Clinical Medical College of Lanzhou University, Lanzhou, China

**Keywords:** Endometrial cancer, nerve-cancer crosstalk, immune infiltration, biomarker, neural-related genes (NRGs)

## Abstract

Endometrial cancer (EC) is the most common gynecologic malignancy, and its incidence has been increasing every year. Nerve signaling is part of the tumor microenvironment and plays an active role in tumor progression and invasion. However, the relationship between the expression of neural-related genes (NRGs) and prognosis in endometrial cancer remains unknown. In this study, we obtained RNA sequencing data of EC from The Cancer Genome Atlas (TCGA). Endometrial cancer was classified into two subtypes based on the expression of neural-associated genes (NRGs), with statistical differences in clinical stage, pathological grading, and prognosis. A prognostic prediction model was established by LASSO-Cox analysis, and the results showed that high expression of NRGs was associated with poor survival prognosis. Further, CHRM2, GRIN1, L1CAM, and SEMA4F were found to be significantly associated with clinical stage, immune infiltration, immune response, and important signaling pathways in endometrial cancer. The reclassification of endometrial cancer based on NRG expression would be beneficial for future clinical practice. The genes CHRM2, GRIN1, L1CAM, and SEMA4F might serve as potential biomarkers of EC prognosis.

## Introduction

In 2020, endometrial cancer has been the sixth most frequent cancer in women worldwide, with 417,000 new cases and 97,000 deaths (1, 2). Although the incidence has leveled off in recent years, it still has been increasing at a rate of 1% per year, making it one of the few human cancers with a rising fatality rate (3). The choice of surgery, radiation, hormonal and/or chemotherapy, immunotherapy, and targeted therapy depend on the stage of the disease.

The tumor microenvironment is composed of neuronal cells, tumor cells, fibroblasts, and immune cells. Cancer cells can generate electroactive tissue by connecting with neural synapses, which drives cancer cells to migrate and develop (4, 5). Active crosstalk between nerves and tumor cells was first observed in prostate and gastric cancers (6, 7), but its role in endometrial cancer remains largely unknown. Perineural infiltration is a new metastatic pathway in endometrial cancer. Endometrial cancer cells have been found to migrate along the neuropil *in vitro*, which is associated with DRG and also a risk factor for perineural infiltration (8). Through cytokinesis, sympathetic nerve endings in the uterus release norepinephrine, adenosine triphosphate (ATP), and other molecules with oxytocic and contractile properties. Uterine parasympathetic fibers primarily release acetylcholine to regulate myometrium activity (9). Furthermore, estrogen and progesterone play important roles in reshaping uterine innervation in response to cyclical changes from puberty to menopause.

Using comprehensive genomic analysis of TCGA, Talhouk et al. classified endometrial cancers into four different subgroups: POLE, microsatellite instability, low copy number, and high copy number (10). Clinically, TCGA molecular typing is practical, useful, and beneficial in predicting the prognosis of patients. Here, we reclassified endometrial carcinoma based on the expression of NRGs. The research on the nerve and endometrial cancer crosstalk can assist the identification of the treatment targets for endometrial cancer. As a result, we found two subgroups related to prognosis by clustering endometrial cancer patients in TCGA data based on NRGs. To find prognosis-associated genes, a prognostic model of neural-associated genes was created. In this study, we investigated the relationship between NRGs and endometrial cancer prognosis, clinical staging, pathological grading, signaling pathways, immune infiltration, and immune response in the hopes of assisting future research.

## Materials and methods

### Source and processing of data sets

The Cancer Genome Atlas (TCGA) database (https://Portal.gdc.cancer.gov/repository) was used to obtain raw data from 552 endometrial cancer patients (11). In addition, clinical endometrial cancer data was retrieved, including survival time, survival status, age, grade, and stage information. 42 NRGs were identified from a previous comprehensive review (12).

### Consensus clustering

The chi-square test and the R language package were used to examine the correlations between clustering and clinical features (13). The “ConsensusClusterPlus” package was used to separate endometrial cancer cases into two subgroups (14). The packages “survival” and “survminer” provided survival analysis between subtypes. The “ggplot2” package was used to identify genetic differences between typings (15), while the “pheatmap” tool was used to create heatmaps. The prcomp function in the statistics package was used to perform principal component analysis (PCA) (16).

### Differential expression analysis

The R package Limma (v3.40.2) was used to study mRNA differential expression (17). Adjusted p-values (FDR) were analyzed in TCGA to correct for false-positive results. The screening conditions for differentially expressed mRNA were | log2FC| ≥ 1 and FDR < 0.05. Determining the cutoff value by the median is the most commonly used method for determining the cutoff value. Similarly, the cut-off value is determined by the interquartile range. High GRIN1/L1CAM expression (top 25%) and low GRIN1/L1CAM expression (bottom 25%) were defined. Since the total expression of CHRM2/SEMA4F was relatively low, 50% was used as the cutoff for high and low expression of both genes. The LASSO regression algorithm was used for feature selection, and 10-fold cross validation was used (18).

### Enrichment analysis and ssGSEA analysis

GO enrichment analysis (19) and KEGG enrichment analysis (20) were done by using R packages ClusterProfiler (21). The Cox regression analysis was performed to identify prognostic genes significantly associated with overall survival (OS) in patients with endometrial cancer (p<0.01) (22). Survival curves were constructed using the R packages “survival” and “survminer”. ROC curves were made using the R packages “survivalROC” and “timeROC”. We collected some functional pathways and calculated the functional pathway scores according to the ssGSEA algorithm.

### Analysis of immune infiltrates

The CIBERSORT (23) and EPIC (24) in the R package “immunedeconv” (https://grst.github.io/immunedeconv) were used to analyze immune infiltrates of different subtypes. It was also visualized using the R package (v4.0.3) ggplot2 and pheatmap (15).

### Analyses of immune checkpoint genes

The correlation of neural-related gene expression with 8 commonly used immune checkpoint genes (CD274, CTLA4, HAVCR2, LAG3, PDCD1, PDCD1LC2, SIGLEC15 and TIGIT) was analyzed and visualized using the R package (v4.0.3) ggplot2 (15) and pheatmap.

### Algorithm for Predicting Immune Responses

Treatment response to immune checkpoint inhibitors can be predicted using the TIDE algorithm (25).

### Stemness analysis

The stemless of mRNA was evaluated using the OCLR method (26).

### Statistical analysis

All statistical analyses were performed in R software (v4.0.3) and were statistically significant at P<0.05.

## Results

### Identification of EC subtypes based on neural-related genes

**Figure 1:**
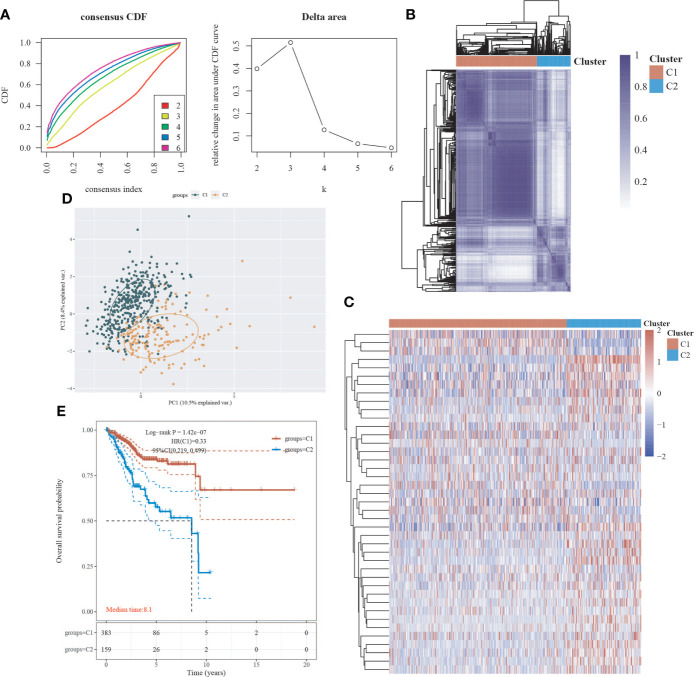
Classification of EC subtypes based on neural-related genes (NRGs). **(A)** Cumulative distribution function (CDF) curve and delta area curve of consensus clustering. **(B)** Heatmap of consensus clustering. Rows and columns represent samples, and different colors represent different categories. **(C)** Heatmap of neural-related gene expression in different subtypes of EC. High expression is represented by red, whereas low expression is represented by blue. **(D)** Principal component analysis (PCA) is a method of analyzing data. **(E)** Survival curves based on the Kaplan-Meier method. Different subgroups’ overall survival curves.

**Figure 2:**
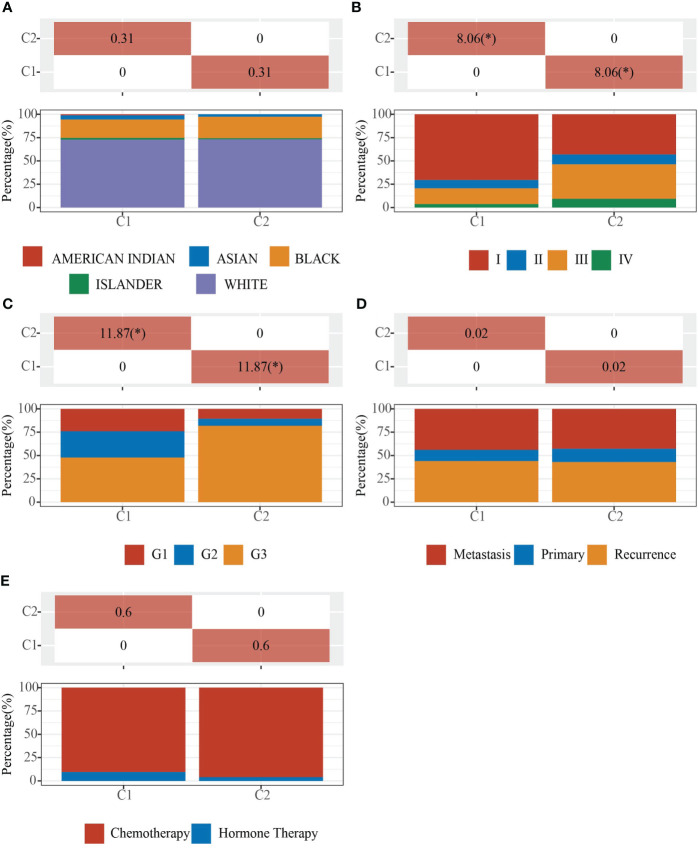
Clinical characteristics of C1 and C2. Demonstration of the proportion of different clinical features in different subgroups. **(A)** Ethnicity. **(B)** Stage. **(C)** Grade. **(D)** Primary, Recurrence and Metastasis. **(E)** Chemotherapy and Hormone Therapy. The * means that the difference in clinical features between the two groups is statistically significant (p<0.05).

We obtained EC data from the TCGA database to explore the relationship between 42 neural-related genes and endometrial cancer. To identify the subtypes, we used the R package ConsensusCluster Plus and two clusters showed up in the result: cluster I (C1) and cluster II (C2) (**Figures 1A-C**). C1 and C2 were found to be well split into two subgroups using principal component analysis (**Figure 1D**). From the retrospective 10-year clinical follow-up study, the overall survival (OS) rates of the two groups were statistically different (P<0.05), with C1 having a greater OS than C2 (**Figure 1E**). In addition, there was a statistically significant difference between C1 and C2 in terms of clinical staging and pathological grading (P<0.05). (**Figure 2**) (**Table 1**).

**Table 1:** Clinical characteristics of C1 and C2.

C1 vs. C2				
	Characteristics	C1	C2	P-value
Status	Alive	339	113	
	Dead	44	47	7.00E-07
Age	Mean (SD)	62.3 (11.5)	67.9 (9.1)	
	Median [MIN, MAX]	62 [31,89]	67 [39,90]	0
Gender	FEMALE	383	160	
Race	AMERICAN INDIAN	4		
	ASIAN	16	4	
	BLACK	71	35	
	ISLANDER	7	2	
	WHITE	261	111	0.495
Stage	I	3		
	IA	136	31	
	IB	111	33	
	IC	20	5	
	II	21	11	
	IIA	4	2	
	IIB	9	4	
	III	1	1	
	IIIA	22	18	
	IIIB	5	1	
	IIIC	18	14	
	IIIC1	10	12	
	IIIC2	9	13	
	IV	3	1	
	IVA	2	1	
	IVB	9	13	8.76E-09
Grade	G1	89	9	
	G2	108	12	
	G3	183	131	
	High Grade	3	8	7.96E-15
new_tumor_event_type	Metastasis	22	15	
	Primary	6	5	
	Recurrence	22	15	0.953
Radiation_therapy	Non-radiation	12	7	
	Radiation	19	12	1
History_of_neoadjuvant_treatment	Neoadjuvant	1	1	
	No neoadjuvant	382	159	1
Therapy_type	Chemotherapy	95	73	
	Chemotherapy:	1	1	
	Chemotherapy::Other. specify in notes:Targeted Molecular therapy	1		
	Chemotherapy:Hormone Therapy	1	5	
	Chemotherapy:Targeted Molecular therapy	2	2	
	Hormone Therapy	10	3	0.251

### Differential expression analysis and enrichment analysis of C1 and C2

**Figure 3:**
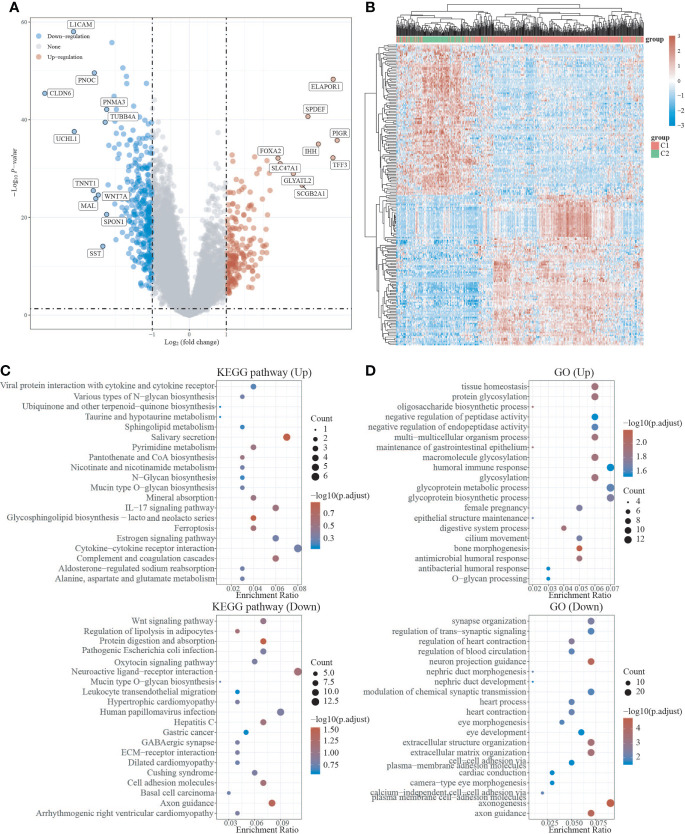
Differential expression and enrichment analysis of C1 and C2. **(A)** Volcano plots of C1 and C2 differentially expressed genes. Blue represents genes with high expression, red represents genes with low expression, and gray represents genes without differential expression. **(B)** Heat map of differential expression. **(C)** C1 activates or suppresses the KEGG pathway when compared to C2. **(D)** C1 activates or suppresses the GO pathway in comparison to C2.

We used the R package Limma to search differentially expressed genes (DEGs) in C1 and C2, with FC>2 and P<0.05 as screening criteria. When compared to C2, C1 showed 209 up-regulated DEGs and 325 down-regulated DEGs. In C1, 24 DEGs were down-regulated (e.g. SEMA4F), 8 DEGs were up-regulated (e.g. ADRB2), and 10 DEGs were unregulated (e.g. GDNF) compared to C2 (**Figures 3A, B**, **S1**). To investigate the activated or suppressed signaling pathways in C1 and C2, KEGG and GO analyses were used (**Figures 3C, D**). KEGG analysis showed that compared to C2, C1 displayed the activation of tumor suppressor pathways like estrogen signaling pathway, ferroptosis, IL-17 signaling pathway, and amino acid metabolic pathway, with the suppression of cancer-associated signaling pathways such as gastric cancer, basal cell carcinoma, Wnt signaling pathway, and neurotransmission. Also, C1 demonstrated the inhibitition of multisystem diseases such as cardiomyopathy, hepatitis C, cushing’s syndrome, human papillomavirus infection, and pathogenic escherichia coli infection. In addition, C1 inhibited lipolysis in adipocytes and oxytocin signaling pathway associated with endometrial carcinogenesis (**Figure 3C**). GO analysis suggested that compared to C2, C1 induced activation of the processes including humoral immune response and antibacterial humoral response, with the inhibition of synapse organization, neuron projection guidance, modulation of chemical synaptic transmission, extracellular matrix organization, cell−cell adhesion, which are generally recognized to be key processes that promote cancer growth and metastasis (**Figure 3D**).

### Immune status analysis of C1 and C2

**Figure 4:**
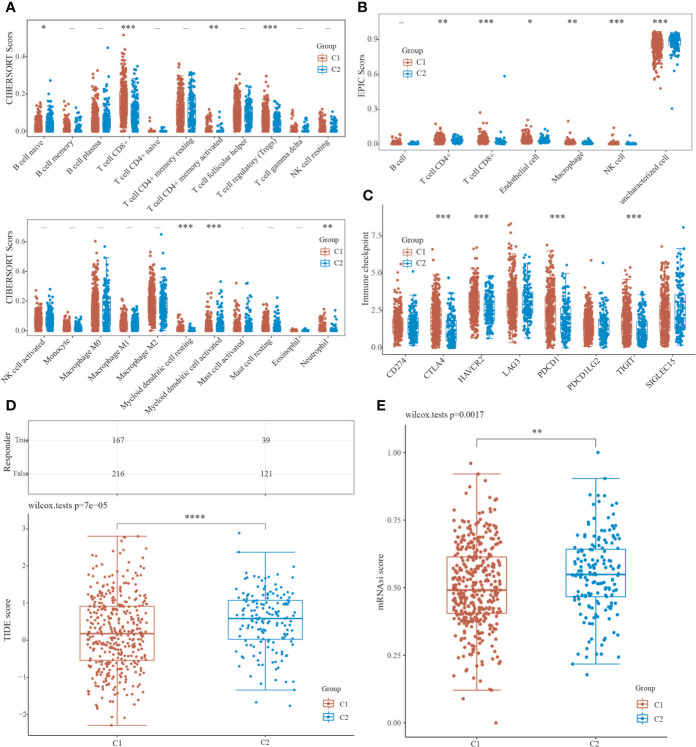
Analysis of C1 and C2 immune infiltration, immunological response, and stemness. **(A, B)** CIBERSORT and EPIC scores reveal a difference in immune cell infiltration between C1 and C2. **(C)** Immune checkpoint-associated genes are expressed differently in C1 and C2. **(D)** TIDE scores for C1 and C2 groups were compared using the TIDE algorithm for predicting cancer immune response. **(E)** The difference in stemness index between C1 and C2 as calculated by the OCLR method. (*P < 0.05, **P < 0.01, ***P < 0.001, ****P < 0.0001).

We used the immunedeconv R package to assess immune infiltration of C1 and C2. the CIBERSORT showed that there were statistically significant differences between C1 and C2 in B cell naive (P<0.05), CD8+ T cells (P<0.001), T cell CD4+ memory activated (P<0.01), T cell regulation (Tregs) (P<0.001), myeloid dendritic cell resting(P<0.001), myeloid dendritic cell activated (P<0.001), and neutrophil (P<0.01), indicating that C2 exhibited stronger immunosuppression compared to C1 (**Figure 4A**). The EPIC further confirmed that C1 and C2 showed stronger immunosuppression in terms of T cell CD4+ (P<0.01) and T cell CD8+ (P<0.001) with statistically significant differences (**Figure 4B**). We also used the ggplot2 and pheatmap R packages to analyze the ICG of both EC subtypes, and the study showed that CTLA4, HAVCR2, PDCD1, and TIGIT expression were increased in C1 (P<0.001) compared to C2 (**Figure 4C**), implying that immunotherapy may be more effective. Furthermore, we utilized the TIDE algorithm to predict cancer immune response, and the findings revealed that the C2 group had a higher TIDE score than the C1 group, with a significant difference (P=7e-05), indicating that the C1 group may benefit more from immune checkpoint inhibitor therapy (ICBs) (**Figure 4D**). The OCLR algorithm revealed that C2 had a greater stemness index than C1, with a statistical difference (P=0.0017), indicating that the C2 group had a higher degree of cancer progression, which could help identify novel targets for anticancer drugs (**Figure 4E**).

### Prognostic analysis of neural-related gene expression in EC

**Figure 5:**
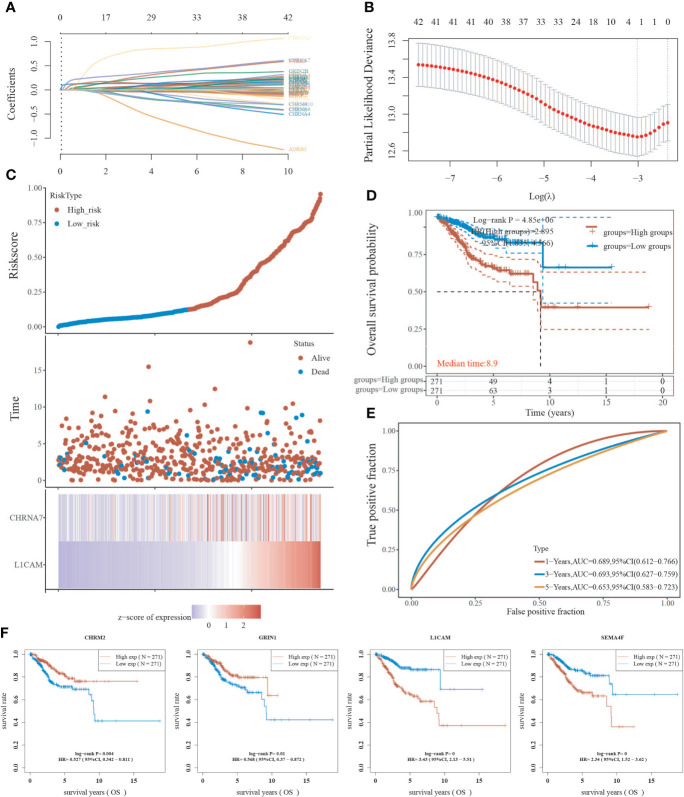
Analysis of neural-associated gene expression in EC for prognosis. **(A)** Coefficients of 42 neural-associated genes represented by λ parameter. **(B)** LASSO COX regression model was used to draw the partial likelihood deviance versus log(λ). **(C)** The correlation between risk and survival. A curve, scatter plot, and heatmap are all used to represent the data. **(D)** Curves of overall survival for high and low risk groups. **(E)** The receiver operating characteristic (ROC) analysis of risk scores. At 1, 3, and 5 years, the area under the curve (AUC) was 0.900, 0.919, and 0.939, respectively. **(F)** A univariate Cox analysis was used to look for genes linked to endometrial cancer prognosis.

Using the LASSO regression technique, we attempted to determine a link between NRGs and EC prognosis. High expression of neurologically linked genes was shown to be associated with a poor prognosis (P<0.05) (**Figures 5A-D**). The expression of 42 NRGs may be a prognostic biomarker for EC patients: the area under the curve (AUC) for 1 year, 3 years, and 5 years was 0.689, 0.693, and 0.653 respectively (**Figure 5E**). Individual prognostic analysis revealed that four of the 42 NRGs were statistically different and linked with EC prognosis (P<0.01) (**Figure 5F**). CHRM2 and GRIN1 were shown to be positively related to EC prognosis, while L1CAM and SEMA4F were found to be adversely related (**Figure 5F**). These results suggest that the expression of neural-related genes including CHRM2, GRIN1, L1CAM, and SEMA4F may be potential biomarkers of EC prognosis.

### Correlation of CHRM2/GRIN1/L1CAM/SEMA4F expression with clinical characteristics

Based on RNA sequencing (RNA seq) and clinical data from the TCGA database, we divided the expression of CHRM2, GRIN1, L1CAM, and SEMA4F into high and low expression groups, and defined the cutoff points for high expression of GRIN1 and L1CAM (top 25%) and low expression of GRIN1 and L1CAM (bottom 25%). Because the total expression of CHRM2 and SEMA4F was low, the cut-off point for high and low expression of both genes was set at 50%. The results showed that the high expression of CHRM2 was negatively correlated with EC grading and staging (**Figure S2A**) (**Table 2**); the high expression of GRIN1 was negatively correlated with EC grading and staging (**Figure S2B**) (**Table 3**); the high expression of L1CAM was positively correlated with EC tumor grading and staging (**Figure S2C**) (**Table 4**); the high expression of SEMA4F was positively correlated with EC tumor grading and staging (**Figure S2D**) (**Table 5**).

**Table 2:** Clinical characteristics of CHRM2 high expression group and CHRM2 low expression group.

	Characteristics	CHRM2 High	CHRM2 Low	P-value
Status	Alive	240	212	
	Dead	32	59	0.003
Age	Mean (SD)	62.8 (10.9)	65.2 (11.3)	
	Median [MIN, MAX]	62 [34,90]	66 [31,90]	0.015
Gender	FEMALE	272	271	
Race	AMERICAN INDIAN	2	2	
	ASIAN	13	7	
	BLACK	43	63	
	ISLANDER	5	4	
	WHITE	201	171	0.11
Stage	I	2	1	
	IA	88	79	
	IB	79	65	
	IC	14	11	
	II	22	10	
	IIA	2	4	
	IIB	8	5	
	III		2	
	IIIA	21	19	
	IIIB	1	5	
	IIIC	10	22	
	IIIC1	9	13	
	IIIC2	6	16	
	IV	1	3	
	IVA	1	2	
	IVB	8	14	0.001
Grade	G1	66	32	
	G2	75	45	
	G3	130	184	
	High Grade	1	10	6.79E-07
new_tumor_event_type	Metastasis	21	16	
	Primary	6	5	
	Recurrence	16	21	0.489
Radiation_therapy	Non-radiation	2	17	
	Radiation	17	14	0.005
History_of_neoadjuvant_treatment	No neoadjuvant	272	269	
	Neoadjuvant		2	0.477
Therapy_type	Chemotherapy	69	99	
	Chemotherapy::Other. specify in notes:Targeted Molecular therapy	1		
	Chemotherapy:Hormone Therapy	2	4	
	Chemotherapy:Targeted Molecular therapy	2	2	
	Hormone Therapy	7	6	
	Chemotherapy:		2	0.55

**Table 3:** Clinical characteristics of GRIN1 high expression group and GRIN1 low expression group.

	Characteristics	GRIN1 High	GRIN1 Low	P-value
Status	Alive	123	103	
	Dead	13	33	0.002
Age	Mean (SD)	62 (11.2)	66.4 (9.7)	
	Median [MIN, MAX]	62 [34,89]	67 [33,90]	0.001
Gender	FEMALE	136	136	
Race	ASIAN	4	5	
	BLACK	18	35	
	ISLANDER	1	2	
	WHITE	106	82	
	AMERICAN INDIAN		1	0.031
pTNM_stage	I	2		
	IA	44	35	
	IB	47	28	
	IC	5	2	
	II	8	6	
	IIA	2	1	
	IIB	3	3	
	III	1	1	
	IIIA	8	14	
	IIIC	7	10	
	IIIC1	3	10	
	IIIC2	1	11	
	IV	1	1	
	IVA	2	1	
	IVB	2	11	
	IIIB		2	0.008
Grade	G1	32	10	
	G2	40	13	
	G3	64	110	
	High Grade		3	7.52E-09
new_tumor_event_type	Metastasis	10	14	
	Primary	3	5	
	Recurrence	8	10	0.946
Radiation_therapy	Non-radiation	5	4	
	Radiation	8	7	1
History_of_neoadjuvant_treatment	No neoadjuvant	136	135	
	Neoadjuvant		1	
Therapy_type	Chemotherapy	35	63	
	Hormone Therapy	4	2	
	Chemotherapy:		2	
	Chemotherapy:Hormone Therapy		1	
	Chemotherapy:Targeted Molecular therapy		1	0.278

**Table 4:** Clinical characteristics of L1CAM high expression group and L1CAM low expression group.

	Characteristics	L1CAM High	L1CAM Low	P-value
Status	Alive	97	125	
	Dead	39	11	1.17E-05
Age	Mean (SD)	68.4 (9.1)	61.2 (11.3)	
	Median [MIN, MAX]	68 [49,90]	61 [34,87]	0
Gender	FEMALE	136	136	
Race	AMERICAN INDIAN	1	1	
	ASIAN	4	6	
	BLACK	39	28	
	ISLANDER	1	2	
	WHITE	80	94	0.474
Stage	I		1	0
	IA	23	50	
	IB	24	44	
	IC	5	6	
	II	11	6	
	IIA	4		
	IIB	3	5	
	IIIA	12	10	
	IIIB	2	1	
	IIIC	13	6	
	IIIC1	11	2	
	IIIC2	14	2	
	IV	1		
	IVA	2	1	
	IVB	11	2	3.33E-08
Grade	G1	2	40	
	G2	8	49	
	G3	119	46	
	High Grade	7	1	1.37E-21
new_tumor_event_type	Metastasis	11	6	
	Primary	1	3	
	Recurrence	14	8	0.313
Radiation_therapy	Non-radiation	9	5	
	Radiation	10	6	1
History_of_neoadjuvant_treatment	Neoadjuvant	1		
	No neoadjuvant	135	136	
Therapy_type	Chemotherapy	74	31	
	Chemotherapy:	1	1	
	Chemotherapy:Hormone Therapy	3		
	Chemotherapy:Targeted Molecular therapy	2	2	
	Hormone Therapy	1	5	
	Chemotherapy::Other. specify in notes:Targeted Molecular therapy		1	0.042

**Table 5:** Clinical characteristics of SEMA4F high expression group and SEMA4F low expression group.

	Characteristics	SEMA4F High	SEMA4F Low	P-value
Status	Alive	212	240	
	Dead	60	31	0.001
Age	Mean (SD)	65.2 (11.2)	62.8 (10.9)	
	Median [MIN, MAX]	64 [33,90]	63 [31,87]	0.013
Gender	FEMALE	272	271	
Race	ASIAN	6	14	
	BLACK	60	46	
	ISLANDER	3	6	
	WHITE	187	185	
	AMERICAN INDIAN		4	0.043
Stage	I	1	2	
	IA	81	86	
	IB	59	85	
	IC	13	12	
	II	21	11	
	IIA	4	2	
	IIB	7	6	
	III	1	1	
	IIIA	19	21	
	IIIB	4	2	
	IIIC	20	12	
	IIIC1	14	8	
	IIIC2	11	11	
	IV	2	2	
	IVA	3		
	IVB	12	10	0.035
Grade	G1	26	72	
	G2	56	64	
	G3	182	132	
	High Grade	8	3	2.97E-07
new_tumor_event_type	Metastasis	24	13	
	Primary	6	5	
	Recurrence	23	14	0.825
Radiation_therapy	Non-radiation	15	4	
	Radiation	17	14	0.155
History_of_neoadjuvant_treatment	No neoadjuvant	272	269	
	Neoadjuvant		2	
Therapy_type	Chemotherapy	91	77	
	Chemotherapy:	1	1	
	Chemotherapy:Hormone Therapy	3	3	
	Chemotherapy:Targeted Molecular therapy	1	3	
	Hormone Therapy	4	9	
	Chemotherapy::Other. specify in notes:Targeted Molecular therapy		1	0.428

### The biological significance of CHRM2/GRIN1/L1CAM/SEMA4F in EC

We classified the EC data in the TCGA database into groups based on the expression levels of four neural-related genes: CHRM2, GRIN1, L1CAM, and SEMA4F, and performed GO and KEGG analyses on each group. The definitions of high and low expression were the same as those mentioned previously. We gathered some functional pathways and used the ssGSEA algorithm to calculate functional pathway scores.

**Figure 6:**
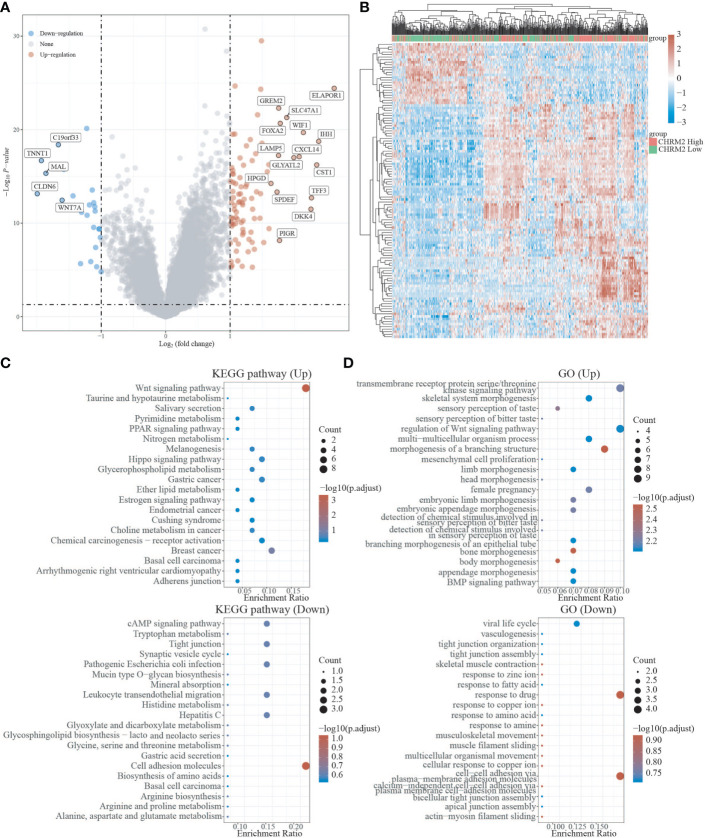
Differential expression and enrichment analysis of CHRM2 high and low expression groups. **(A)** The volcano plot shows the differential gene expression of CHRM2 high expression group and CHRM2 low expression group was drawn with fold-change values and adjusted P. **(B)** Differential gene expression showed by heatmap (only 50 genes were displayed because of the large quantity of the genes); **(C, D)** KEGG and GO analysis showed the upregulated/downregulated pathways of the CHRM2 high expression group compared with the low expression group. When P<0.05 or FDR<0.05 is considered to be enriched to a meaningful pathway.

**Figure 7:**
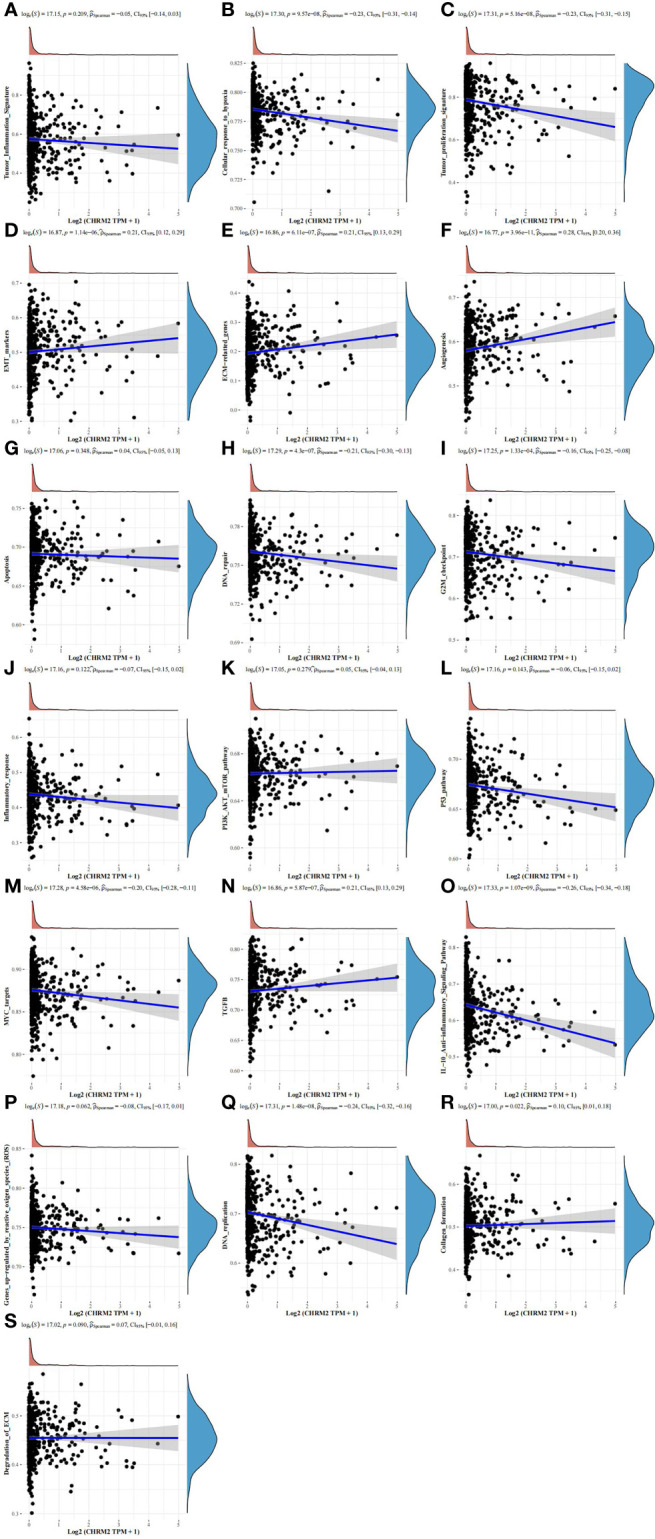
Analysis of the correlation between CHRM2 and 19 pathways using the Spearman, ssGSEA algorithm. (Statistically significant difference at P < 0.05). **(A, G, J–L, P, S)** The ssGSEA analysis showed that the CHRM2 gene was not statistically correlated with tumor inflammation signature, apoptosis, infammatory response, PI3K-AKT-MTOR pathway, p53 pathway, gene up-regulated by reactive oxigen specis (ROS) and degradation of ECM. **(B, C, H, I, M, O, Q)** CHRM2 gene was negatively associated with cellular response to hypoxia, tumor proliferation signature, DNA repair, G2M checkpoint, MYC targets, IL-10 anti-inflammatory signaling pathway and DNA replication. **(D–F, N, R)** CHRM2 gene was positively associated with EMT markers, ECM-related genes, angiogenesis, TGFB and collagen formation.

In the EC with high CHRM2 expression, 92 genes were upregulated and 24 genes were downregulated (FC>2, P<0.05) (**Figures 6A, B**). Compared with the CHRM2 low expression group, the CHRM2 highly-expressed group contained upregulation of tumor-promoting pathways including endometrial cancer, Wnt signaling pathway, and estrogen signaling pathway. It also inhibits endometrial carcinogenesis and metastasis by activating the PPAR signaling system, suppressing the cAMP signaling pathway, cell adhesion molecules, vasculogenesis, amino acid synthesis and metabolism, and mucin-type O−glycan biosynthesis, among other things (**Figures 6C, D**). ssGSEA analysis showed that the CHRM2 gene was negatively associated with cellular response to hypoxia, tumor proliferation signature, DNA repair, G2M checkpoint, MYC targets, IL-10 anti-inflammatory signaling pathway, DNA replication, and positively associated with EMT markers, ECM-related genes, angiogenesis, TGFB, collagen formation (**Figure 7**).

In the EC with high GRIN1 expression, 465 genes were upregulated and 264 genes were downregulated (FC>2, P<0.05) (**Figures S3A**, **S3B**). Compared with the GRIN1 low expression group, the GRIN1 high expression group inhibited the Wnt signaling pathway, signaling pathway regulating pluripotency of stem cells, cell adhesion molecules, hepatocellular carcinoma, and gastric cancer. Activation of cancer-related signaling pathways: p53 signaling pathway, cAMP signaling pathway, AMPK signaling pathway, breast cancer, and prostate cancer (**Figures S3C**, **S3D**). ssGSEA analysis showed that GRIN1 was negatively associated with cellular response to hypoxia, tumor proliferation signature, apoptosis, DNA repair, G2M checkpoint, inflammatory response, MYC targets, TGFB, IL-10 anti-inflammatory signaling pathway, DNA replication, collagen formation, and ECM degradation (**Figure S4A**).

In the EC with high L1CAM expression, 568 genes were upregulated and 493 genes were downregulated (FC>2, P<0.05) (**Figures S5A**, **S5B**). Compared with the L1CAM low expression group, the L1CAM highly-expressed group is consisted of upregulation of tumor-promoting pathways including PI3K-Akt signaling pathway, MAPK signaling pathway, cell adhesion molecules, and synapse organization. The p53 signaling pathway, Wnt signaling pathway, estrogen signaling pathway, and endometrial cancer pathway were inhibited (**Figures S5C**, **S5D**). ssGSEA analysis showed that L1CAM positively connected with cellular response to hypoxia, tumor proliferation signature, DNA repair, G2M checkpoint, MYC targets, TGFB, IL-10 anti-inflammatory signaling pathway, DNA replication, collagen production, and ECM degradation. reactive oxygen species (ROS) upregulation of genes was found to be negatively linked (**Figure S4B**).

In the EC with high SEMA4F expression, 11 genes were upregulated and 62 genes were downregulated (FC>2, P<0.05) (**Figures S6A**, **S6B**). Compared to the SEMA4F low expression group, The SEMA4F high expression group activated multiple diseases of neurodegeneration, Parkinson’s disease, and Alzheimer’s disease while inhibiting the IL-17 signaling pathway, chemical carcinogenesis, protein-coupled receptor signaling pathway, axoneme assembly, and acute inflammatory response (**Figures S6C**, **S6D**). ssGSEA analysis showed that SEMA4F was negatively correlated with tumor inflammation signature, ECM-related gene, angiogenesis, apoptosis, inflammatory response, P53 pathway, IL-10 anti-inflammatory signaling pathway, genes up-regulated by reactive oxygen species (ROS), tumor proliferation signature, DNA Repair, G2M checkpoint, MYC targets, TGFB, and DNA replication (**Figure S4C**).

The findings imply that the genes CHRM2, GRIN1, L1CAM, and SEMA4F in EC have pro- or oncogenic effects as a result of the combined activity of several signaling pathways influencing tumor growth.

### Correlation between CHRM2/GRIN1/L1CAM/SEMA4F expression and immune infiltration, immune response and stemness

We used the immunedeconv R package to obtain immune infiltration data for high/low expression of CHRM2, GRIN1, L1CAM, and SEMA4F in EC. In this research, the CIBERSORT and EPIC algorithms were applied.

**Figure 8:**
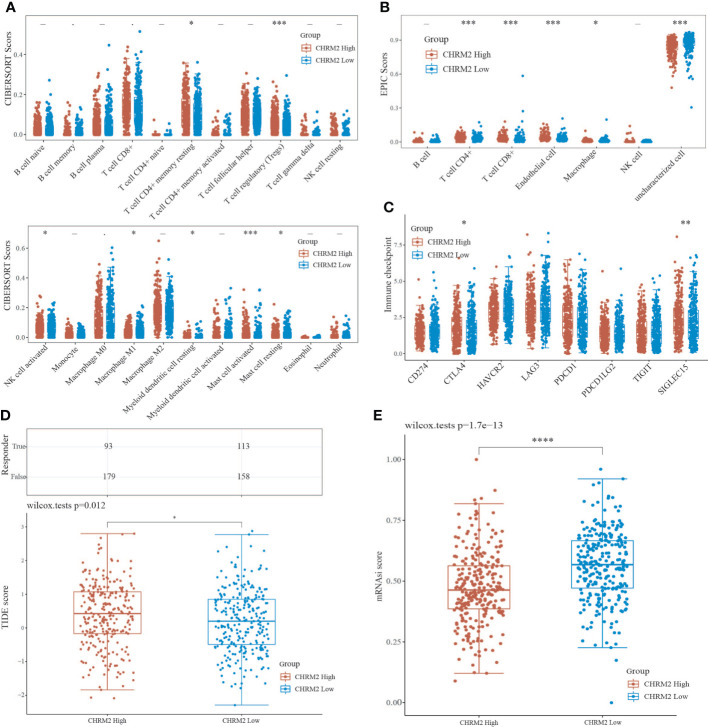
An analysis of immune infiltration, immunological response, and stemness in two groups with high and low CHRM2 expression. **(A, B)** Comparison of CHRM2 high expression group and CHRM2 low expression group in immune infiltration obtained with CIBERSORT and EPIC algorithm; The horizontal axis represents different immune cells, the vertical axis represents the immune scores (*P<0.05, ***P<0 .001). **(C)** Comparison immune checkpoint genes expression in CHRM2 high expression group and CHRm2 low expression group; The horizontal axis represents different immune checkpoint genes, the vertical axis represents the expression level (*P<0.05,**p<0.01). **(D)** Statistical table of immune response and the distribution of immune response scores of the different groups in predict results. (*P<0 .05). **(E)** Comparison of CHRM2 high expression group and CHRM2 low expression group in stemness was exhibited by mRNAsi score with OCLR algorithm (****p<0.0001).

The CIBERSORT algorithm showed that high expression of CHRM2 was positively correlated with T cell CD4+ memory resting (P<0.05) and T cell regulatory (Tregs) (P<0.001), myeloid dendritic cell resting (P<0.05), mast cell activated (P<0.001), and mast cell resting (P<0.05), while it was negatively correlated with NK cell activated (P<0.05), and macrophage M1 (P<0.05) were negatively correlated (**Figure 8A**). The EPIC algorithm showed that high expression of CHRM2 was positively correlated with T cell CD4+ (P<0.001), T cell CD8+ (P<0.001), and endothelial cell (P<0.001), but negatively correlated with macrophage (P<0.05) (**Figure 8B**).

The CIBERSORT algorithm showed that high GRIN1 expression was positively correlated with T cell CD8+ (P<0.01), T cell regulatory (Tregs) (P<0.001), and NK cell resting (P<0.01) compared to low GRIN1 expression (P<0.001), while it was negatively correlated with B cell plasma (P<0.05), NK cell activated (P<0.05), macrophage M2 (P<0.05), and myeloid dendritic cell activated (P<0.05) (**Figure S7A**). The EPIC algorithm showed that high expression of GRIN1 was positively correlated with T cell CD4+ (P<0.001), T cell CD8+ (P<0.001), endothelial cell (P<0.05), but negatively correlated with macrophage (P<0.01) (**Figure S7B**).

The CIBERSORT algorithm showed that high L1CAM expression was positively correlated with T cell follicular helper (P<0.01), NK cell activated (P<0.01), macrophage M1 (P<0.001), and myeloid dendritic cell activated (P<0.01), while it was negatively correlated with T cell CD8+ (P<0.05), T cell CD4+ memory resting (P<0.05), T cell regulatory (Tregs) (P<0.001), NK cell resting (P<0.01), macrophage M2(P<0.05), myeloid dendritic cell resting (P<0.001) and neutrophil (P<0.05) (**Figure S8A**). The EPIC algorithm showed that high expression of L1CAM was positively correlated with B cell (P<0.05) and with T cell CD4+ (P<0.001) and T cell CD8+ (P<0.001), but endothelial cell (P<0.01) negatively (**Figure S8B**).

The CIBERSORT algorithm showed that high SEMA4F expression was positively correlated with B cell naive(P<0.01), myeloid dendritic cell resting(P<0.01), myeloid dendritic cell activated(P<0.001), and mast cell activated(P<0.05), while it was negatively correlated with T cell CD8+(P<0.001), T cell CD4+ memory activated(P<0.01), T cell regulatory (Tregs) (P<0.001) and neutrophil(P<0.05) (**Figure S9A**). The EPIC algorithm showed that high expression of SEMA4F was positively correlated with T cell CD4+ (P<0.01), but macrophage (P<0.01) and NK cell(P<0.001) negatively (**Figure S9B**).

In addition, we analyzed the correlation between ICG and the expression of CHRM2, GRIN1, L1CAM, and SEMA4F. CHRM2 expression was positively correlated with CTLA4 (P<0.05) and SIGLEC15 (P<0.01) (**Figure 8C**); GRIN1 expression was positively correlated with CTLA4 (P<0.05), while negatively correlated with PDCD1LG2 (P<0.01) (**Figure S7C**); L1CAM expression was positively correlated with CD274 (P<0.05), LAG3 (P<0.001) and PDCD1LG2 (P<0.05), while negatively correlated with CTLA4 (P<0.001) (**Figure S8C**). SEMA4F was positively correlated with HAVCR2 (P<0.01), LAG3 (P<0.001), PDCD1 (P<0.001) and TIGIT (P<0.01) (**Figure S8C**), while negatively correlated with CTLA4 (P<0.001), HAVCR2 (P<0.01), LAG3 (P<0.05), PDCD1 (P<0.001), TIGIT (P<0.001) and SIGLEC15 (P <0.05) (**Figure S9C**). The TIDE algorithm revealed that high expression of CHRM2, and L1CAM was linked to poor immune response (**Figures 8D**, **S8D**), whereas high expression of GRIN1 was linked to a positive immunological response (**Figure S7D**). Stem cell scores were lower in the high expression group of CHRM2 (**Figure 8E**) and GRIN1 (**Figure S7E**) than in the low expression group, according to Spearman correlation analysis of OCLR scores, while the inverse was true for L1CAM (**Figure S8E**) and SEMA4F (**Figure S9E**).

### Gene landscape of CHRM2/GRIN1/L1CAM/SEMA4F

**Figure 9:**
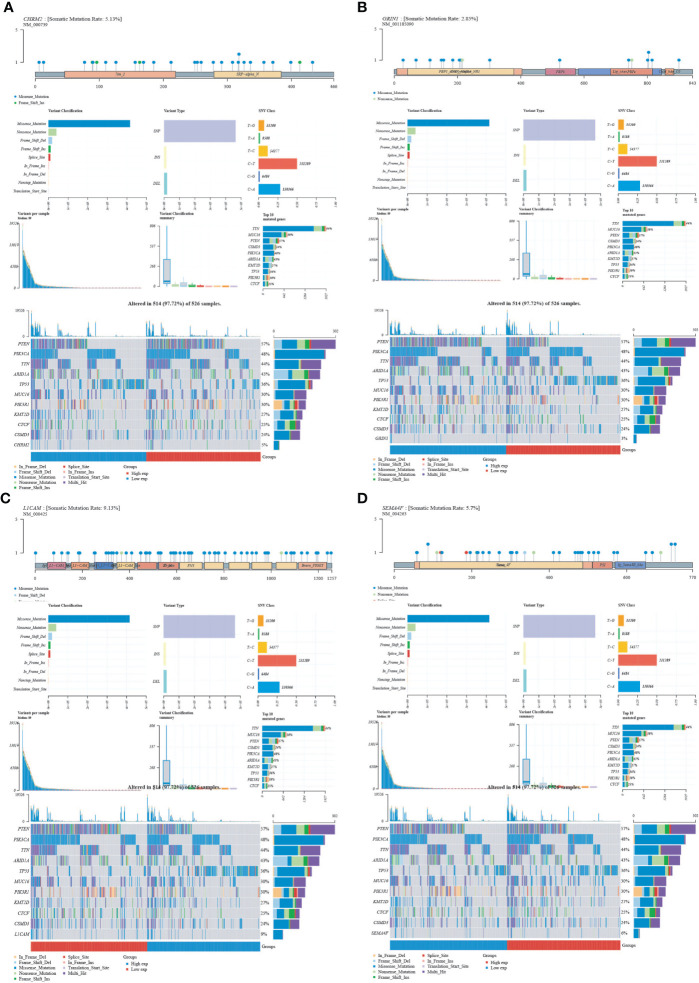
Mutational landscape of CHRM2, GRIN1, L1CAM and SEMA4F. **(A)** CHRM2. **(B)** GRIN1. **(C)** L1CAM. **(D)** SEMA4F.

**Figure 10:**
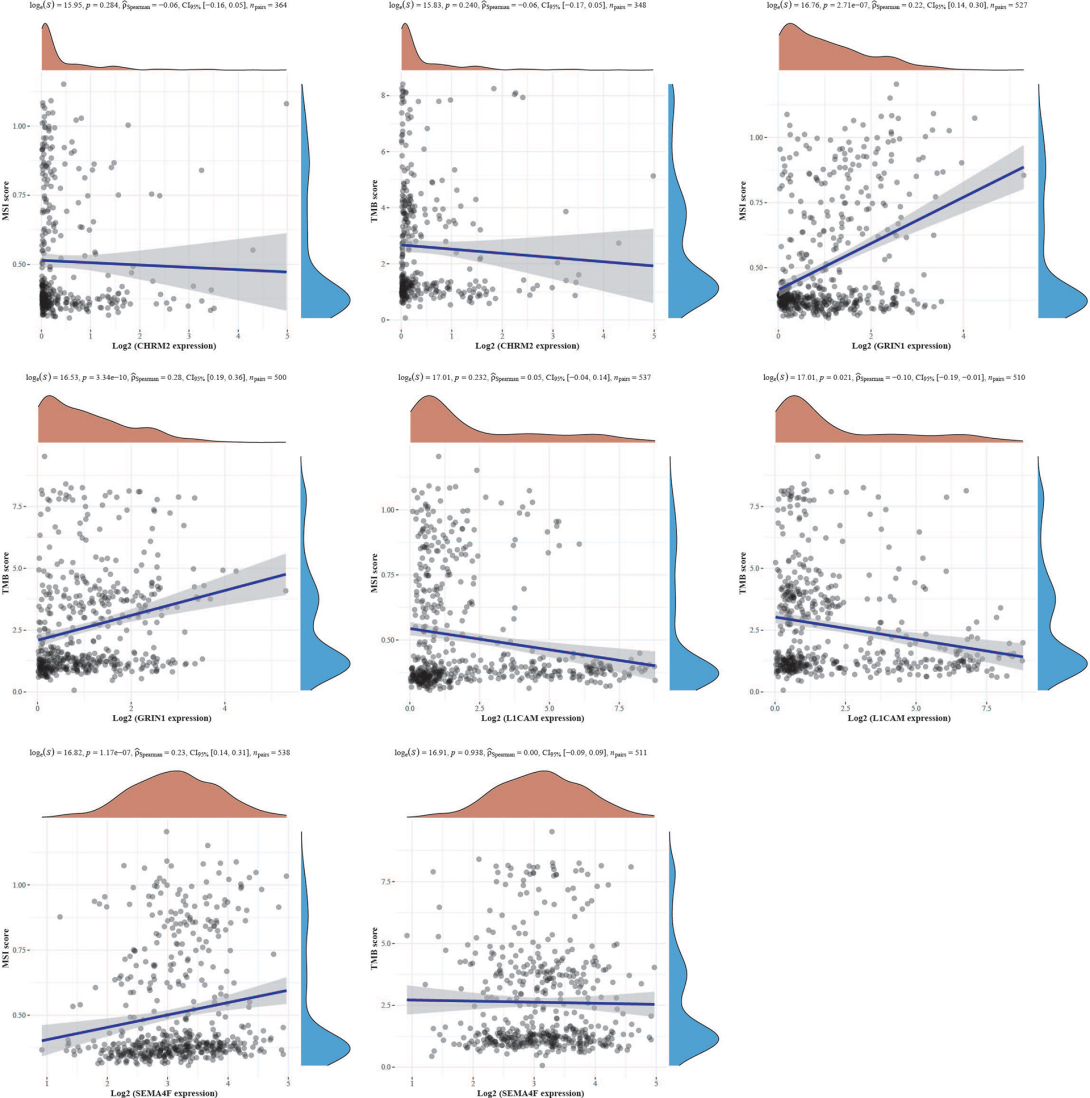
The correlation of CHRM2, GRIN1, L1CAM, and SEMA4F with TMB and MSI using Spearman’s technique. (Statistically significant difference at P < 0.05).

We obtained mutational, transcriptomic, and clinical data of EC patients from the TCGA database and found the highest rate of PETN mutations in EC (57%), with varying degrees of mutations in neuro-oncology-related genes CHRM2, GRIN1, L1CAM, and SEMA4F: L1CAM (9%), SEMA4F (6%), CHRM2 (5%), and GRIN1 (3%) (**Figure 9**). The differences between GRIN1 and tumor mutational load (TMB) and microsatellite instability (MSI) were statistically significant, while L1CAM was negatively connected with TMB and SEMA4F was positively correlated with MSI. The findings imply that GRIN1, L1CAM, and SEMA4F are closely linked to immunotherapy and can respond to immunotherapy characteristics (**Figure 10**).

## Discussion

In the tumor microenvironment, neurons are critical biological components. Denervation and regulation of neurotransmitters for tumor treatment have become hot topics of research in recent years (27). Tumors select neuronal programs to promote their development and progression. The frequency of endometrial cancer has been increasing each year as is the number of patients with endometrial cancer brain metastases (28). Although nerve-cancer crosstalk influences tumor growth, the etiology is yet unknown (7).

The identification of cancer subgroups based on gene expression has proven useful in clinical settings, such as endometrial cancer molecular staging (29). Based on NRGs, we classified endometrial cancer into two subtypes: C1 and C2. Prognosis, clinical-stage, pathological grading, and immunological status were all statistically different between the two subtypes. In comparison to C2, C1 had a lower clinical stage and pathological grade, a better prognosis, better immune activation, stronger immune checkpoint gene expression, and was more suited to immunotherapy. In addition, there were statistical differences in enriched pathways and biological processes between C1 and C2. These data support the link between neural-related genes and endometrial cancer, and they suggest that using NRGs to classify EC subtypes could be clinically effective.

A prognostic model was constructed by LASSO-Cox, and CHRM2, GRIN1, L1CAM, and SEMA4F were identified as EC prognostic-related genes. CHRM2 is a gene encoding muscarinic receptor (mAChR) on neuronal cell membranes, which affects cholinergic activity by influencing the transcription level, mRNA stability, and affinity of the receptor (30). Previous studies have found that CHRM2 is enriched in the PI3K-Akt signaling pathway and its methylation rate rises as a progression of gastric cancer (31). CHRM2 inhibits the invasion and migration of non-small cell lung cancer through the M2R/ERK/Akt/NF-κB axis (32). In the central nervous system (CNS), glutamate receptor subunit 1 (GRIN1) is essential for synaptic transmission and plasticity (33). GRIN1 mutations are linked to schizophrenia, neurodevelopmental delay, epilepsy, and glioma, but no other tumors are linked to them (34–36). For the first time, our findings reveal that CHRM2 and GRIN1 play significant roles in endometrial cancer and are positively related to endometrial cancer prognosis. L1 cell adhesion molecule (L1CAM) is a membrane glycoprotein of the immunoglobulin family (37). Consistent with previous findings, L1CAM plays a key role in EC cancer cell migration and adhesion (38). Furthermore, we discovered that L1CAM was linked to immune cell infiltration and ICG (CD274, LAG3, PDCD1LG2, CTLA4), and we hypothesized that L1CAM may have a regulatory role in the tumor microenvironment, influencing tumor growth and metastasis. SEMA4F is a membrane-bound glycoprotein of the signaling element receptor family that has been linked to cancer in prior research, including being associated to breast cancer development (39), axonogenesis and neurogenesis in prostate cancer (40), and glioma prognosis (41). We propose that SEMA4F is a key regulator of tumor growth, angiogenesis, migration, and apoptosis and that it plays a role in endometrial cancer.

In summary, our study reveals the relevance of neural-related genes to endometrial cancer. Our findings suggest that EC reclassification based on neural-related genes is expected to be translated into clinical applications. The genes CHRM2, GRIN1, L1CAM, and SEMA4F, which are prognostically associated with endometrial cancer, play important roles in immune cell infiltration, immune response and stem cell relevance, clinical features, enriched pathways, and immunotherapy, and are potential biomarkers for EC with significant clinical translational potential. Further investigation can considered to quantify the indicators through tissue specimens and animal experiments to validate them for greater application in the treatment of tumors.

## Data availability statement

The original contributions presented in the study are included in the article/**Supplementary Material**. Further inquiries can be directed to the corresponding authors.

## Author contributions

FC and YZ did the analysis. FC wrote the paper. YD and PL did the data sorting and charting. TQ and WJ conceived the paper. All authors contributed to the article and approved the submitted version.

## Funding

This work was in part supported by the Innovation and Entrepreneurship Talent Project of Lanzhou (2020-RC-52) and the “Innovation Fund” for graduate students of the First Clinical Medical College of Gansu University of Chinese Medicine (LCCX2021011).

## Acknowledgments

We apologize to those colleagues whose important work could not be cited due to space constraints.

## Conflict of Interest

The authors declare that the research was conducted in the absence of any commercial or financial relationships that could be construed as a potential conflict of interest.

## Publisher’s note

All claims expressed in this article are solely those of the authors and do not necessarily represent those of their affiliated organizations, or those of the publisher, the editors and the reviewers. Any product that may be evaluated in this article, or claim that may be made by its manufacturer, is not guaranteed or endorsed by the publisher.

## Supplementary material

Supplementary Figure 1 Comparison of differential expression of 42 neural-related genes between C1 and C2.

Supplementary Figure 2 Comparison of the clinical characteristics of the two groups with high and low expression of CHRM2, GRIN1, L1CAM and SEMA4F. **(A)** CHRM2. **(B)** GRIN1. **(C)** L1CAM. **(D)** SEMA4F.

Supplementary Figure 3 Differential expression and enrichment analysis of GRIN1 high expression group and GRIN1 low expression group.

Supplementary Figure 4 The connection of GRIN1, L1CAM, and SEMA4F with 19 pathways was investigated using the Spearman, ssGSEA algorithm. (S4A) GRIN1.**(A)** GRIN1. **(B)** L1CAM. **(C)** SEMA4F.

Supplementary Figure 5 Differential expression and enrichment analysis of L1CAM high expression group and L1CAM low expression group.

Supplementary Figure 6 Differential expression and enrichment analysis of SEMA4F high expression group and SEMA4F low expression group.

Supplementary Figure 7 Analysis of immune infiltration, immunological response, and stemness in two groups with high and low GRIN1 expression.

Supplementary Figure 8 Analysis of immune infiltration, immunological response, and stemness in two groups with high and low L1CAM expression.

Supplementary Figure 9 Analysis of immune infiltration, immunological response, and stemness in two groups with high and low SEMA4F expression.
