# Supplementary figures and images for: Reclassification of endometrial cancer and identification of key genes based on neural-related genes
Source: Front Oncol. 2022 Sep 23;12:951437. doi: 10.3389/fonc.2022.951437 (PMC9537575; doi:10.3389/fonc.2022.951437)

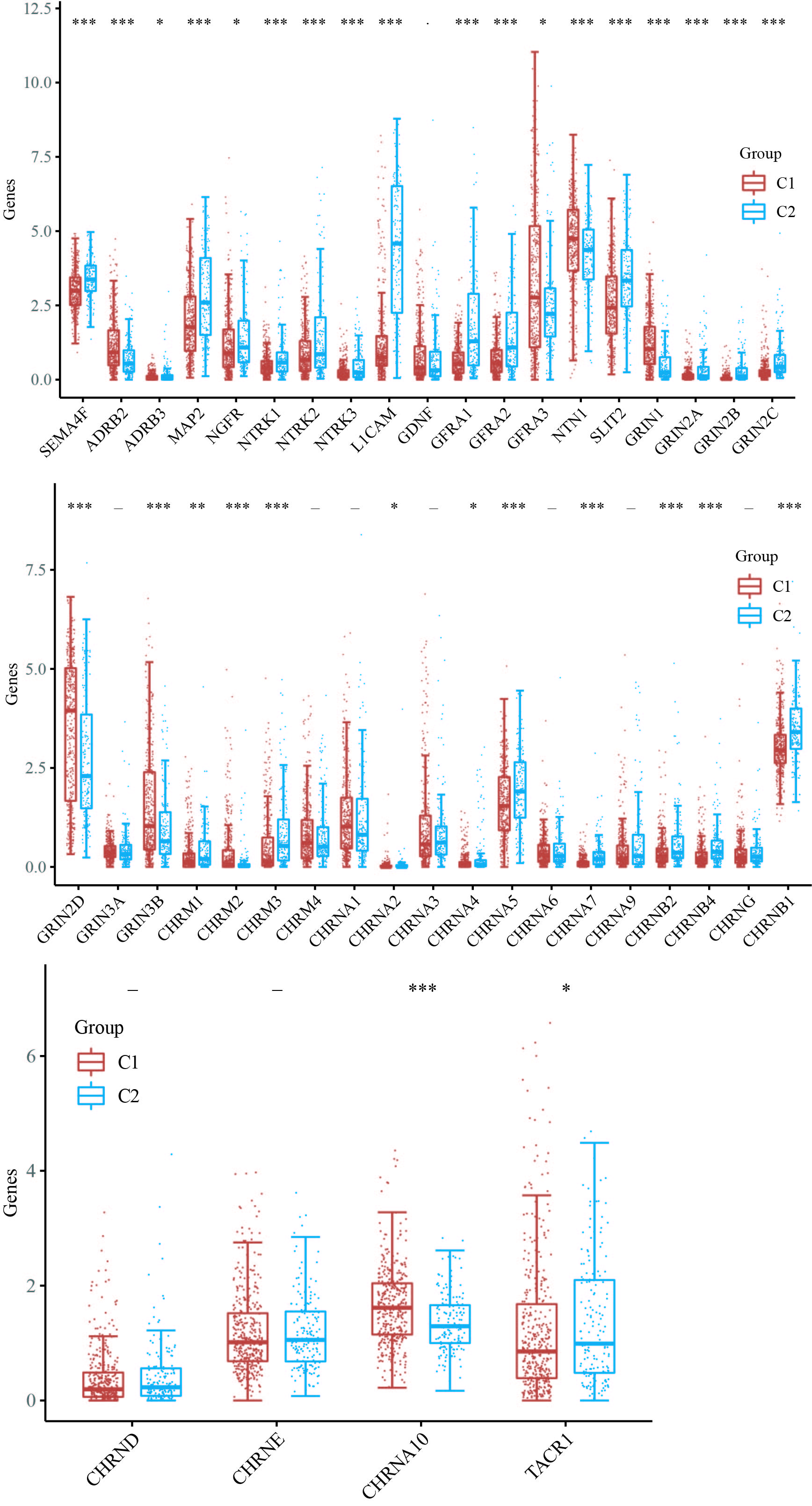

Supplement: Supplementary Figure 1 — Comparison of differential expression of 42 neural-related genes between C1 and C2. [file Image_1.jpg]

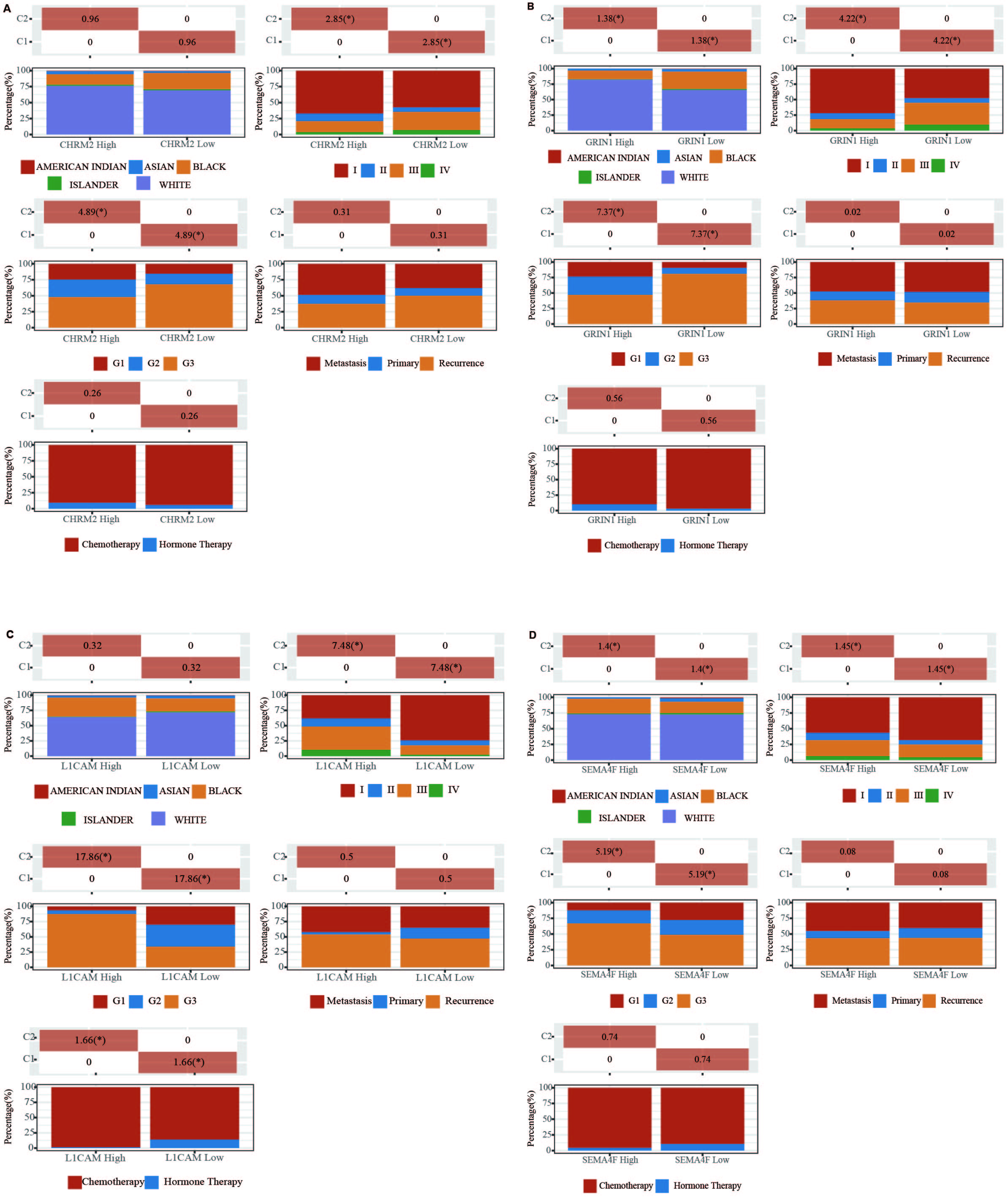

Supplement: Supplementary Figure 2 — Comparison of the clinical characteristics of the two groups with high and low expression of CHRM2, GRIN1, L1CAM and SEMA4F. (A) CHRM2. (B) GRIN1. (C) L1CAM. (D) SEMA4F. [file Image_2.jpg]

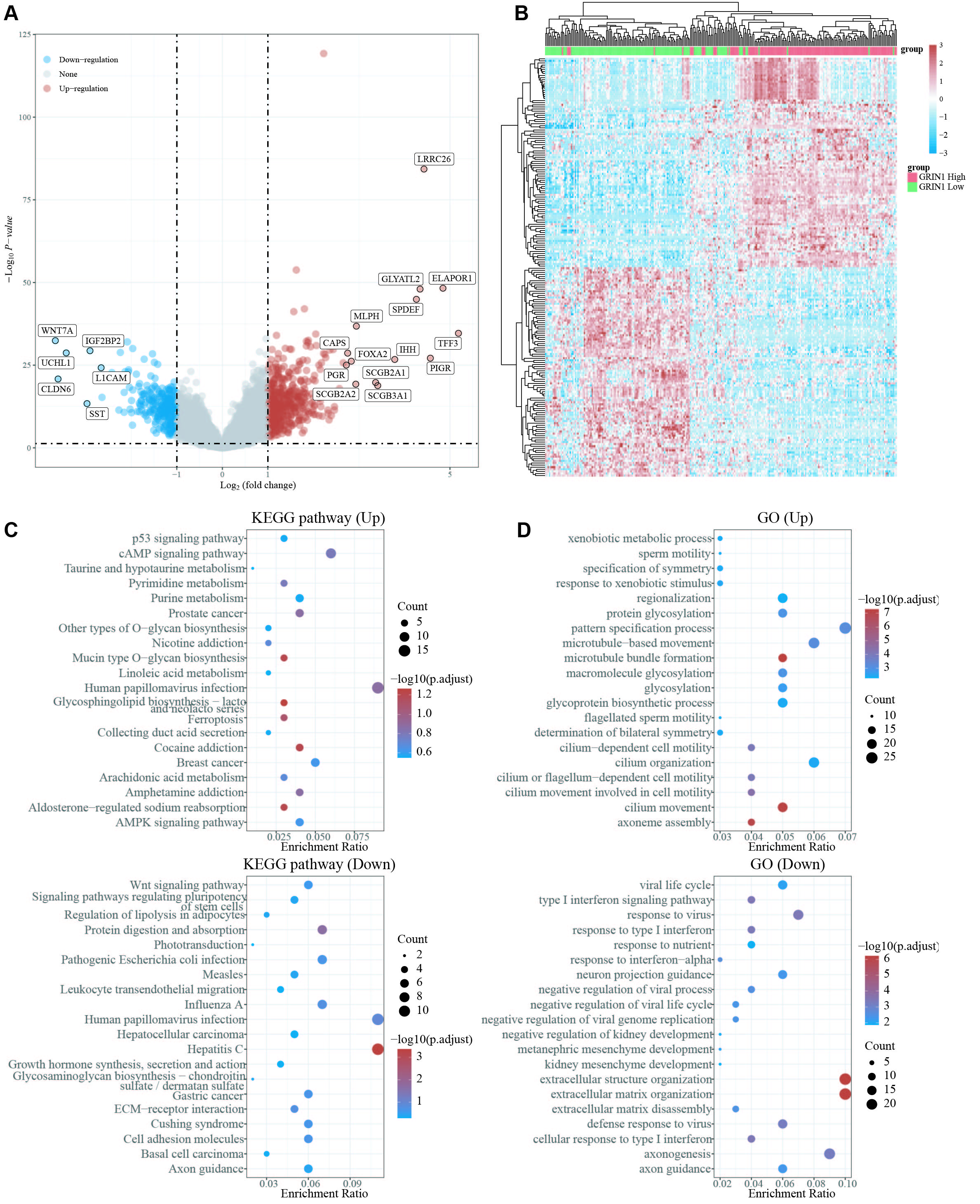

Supplement: Supplementary Figure 3 — Differential expression and enrichment analysis of GRIN1 high expression group and GRIN1 low expression group. [file Image_3.jpg]

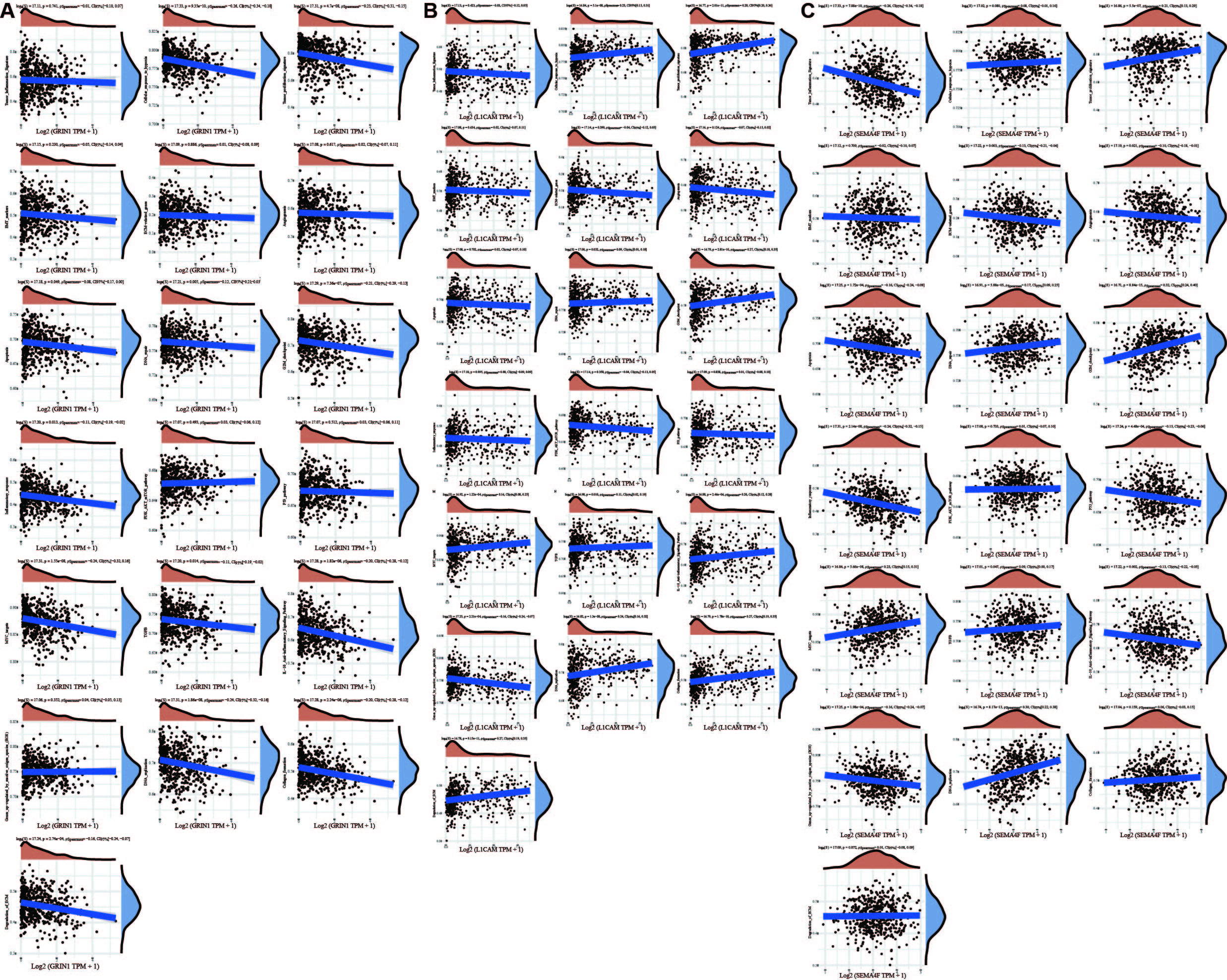

Supplement: Supplementary Figure 4 — The connection of GRIN1, L1CAM, and SEMA4F with 19 pathways was investigated using the Spearman, ssGSEA algorithm. (S4A) GRIN1.(A) GRIN1. (B) L1CAM. (C) SEMA4F. [file Image_4.jpg]

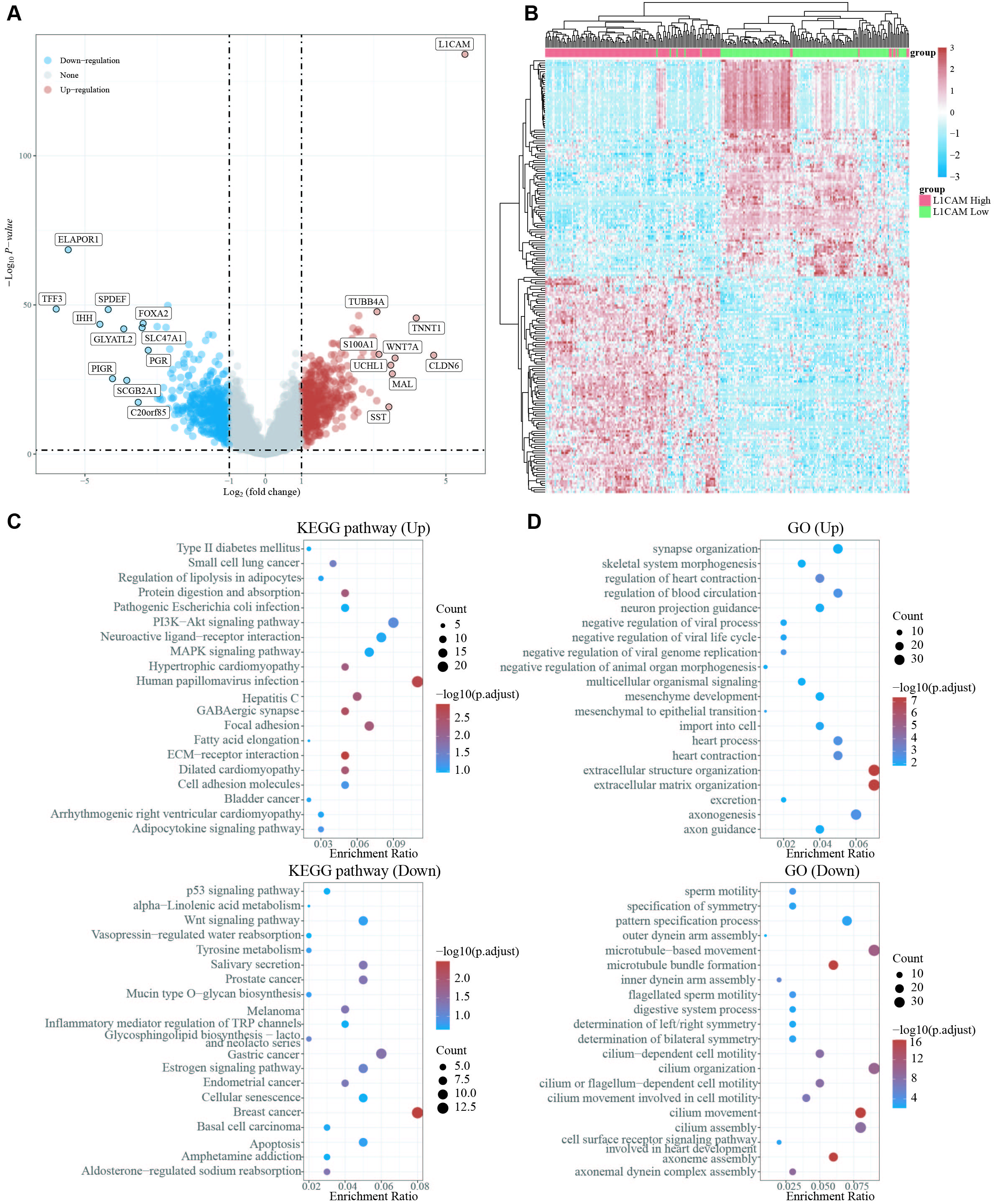

Supplement: Supplementary Figure 5 — Differential expression and enrichment analysis of L1CAM high expression group and L1CAM low expression group. [file Image_5.jpg]

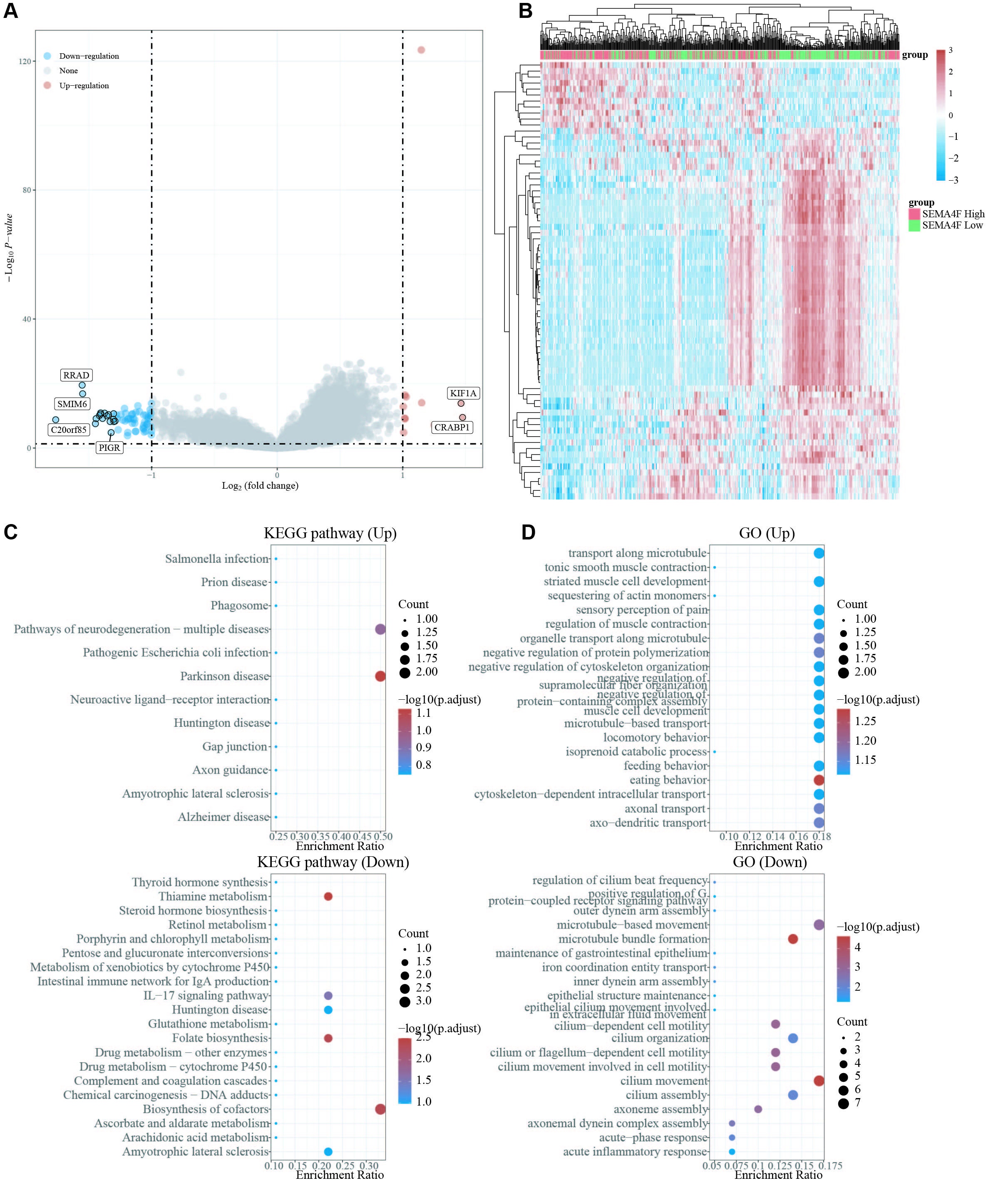

Supplement: Supplementary Figure 6 — Differential expression and enrichment analysis of SEMA4F high expression group and SEMA4F low expression group. [file Image_6.jpg]

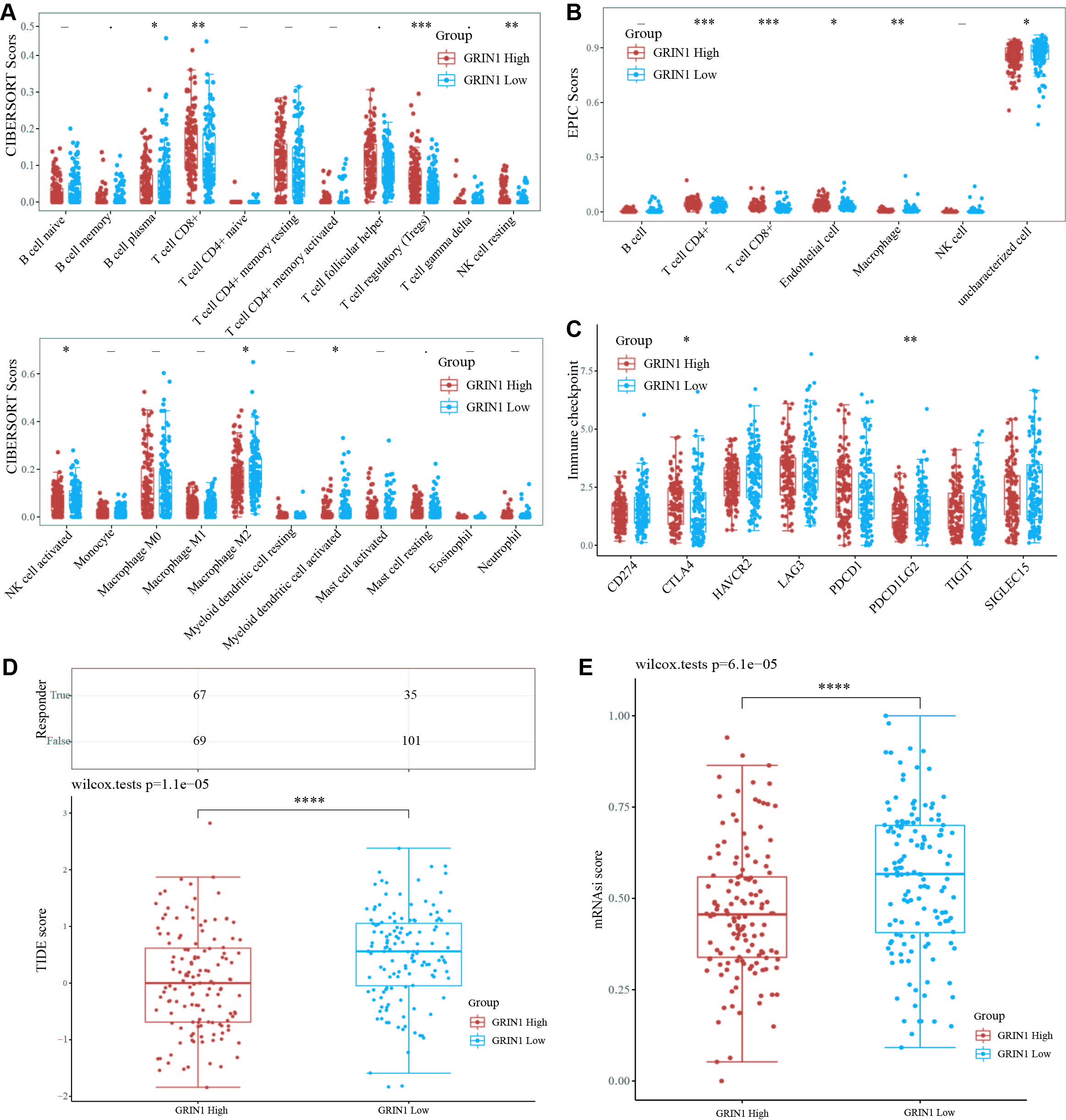

Supplement: Supplementary Figure 7 — Analysis of immune infiltration, immunological response, and stemness in two groups with high and low GRIN1 expression. [file Image_7.jpg]

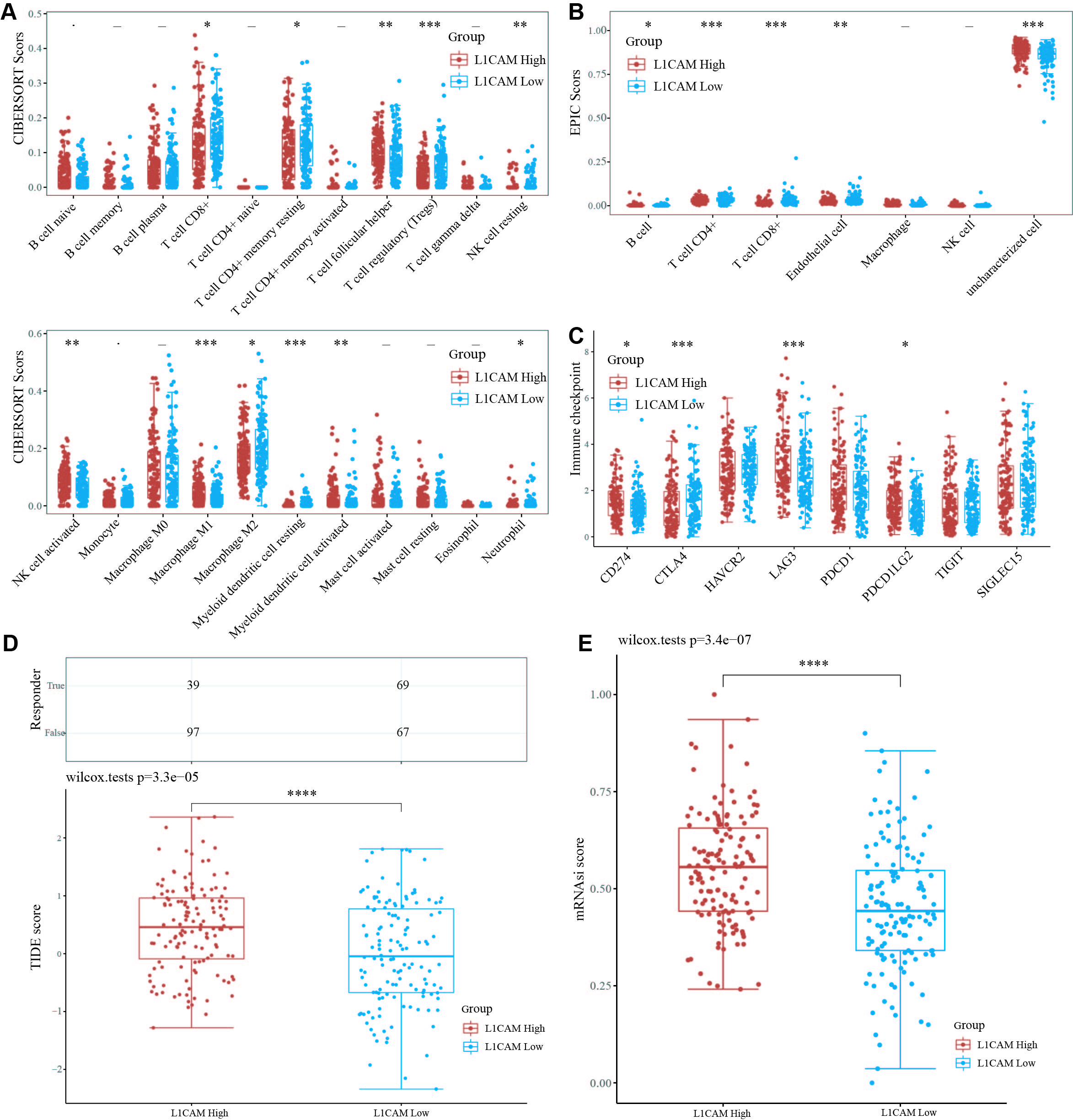

Supplement: Supplementary Figure 8 — Analysis of immune infiltration, immunological response, and stemness in two groups with high and low L1CAM expression. [file Image_8.jpg]

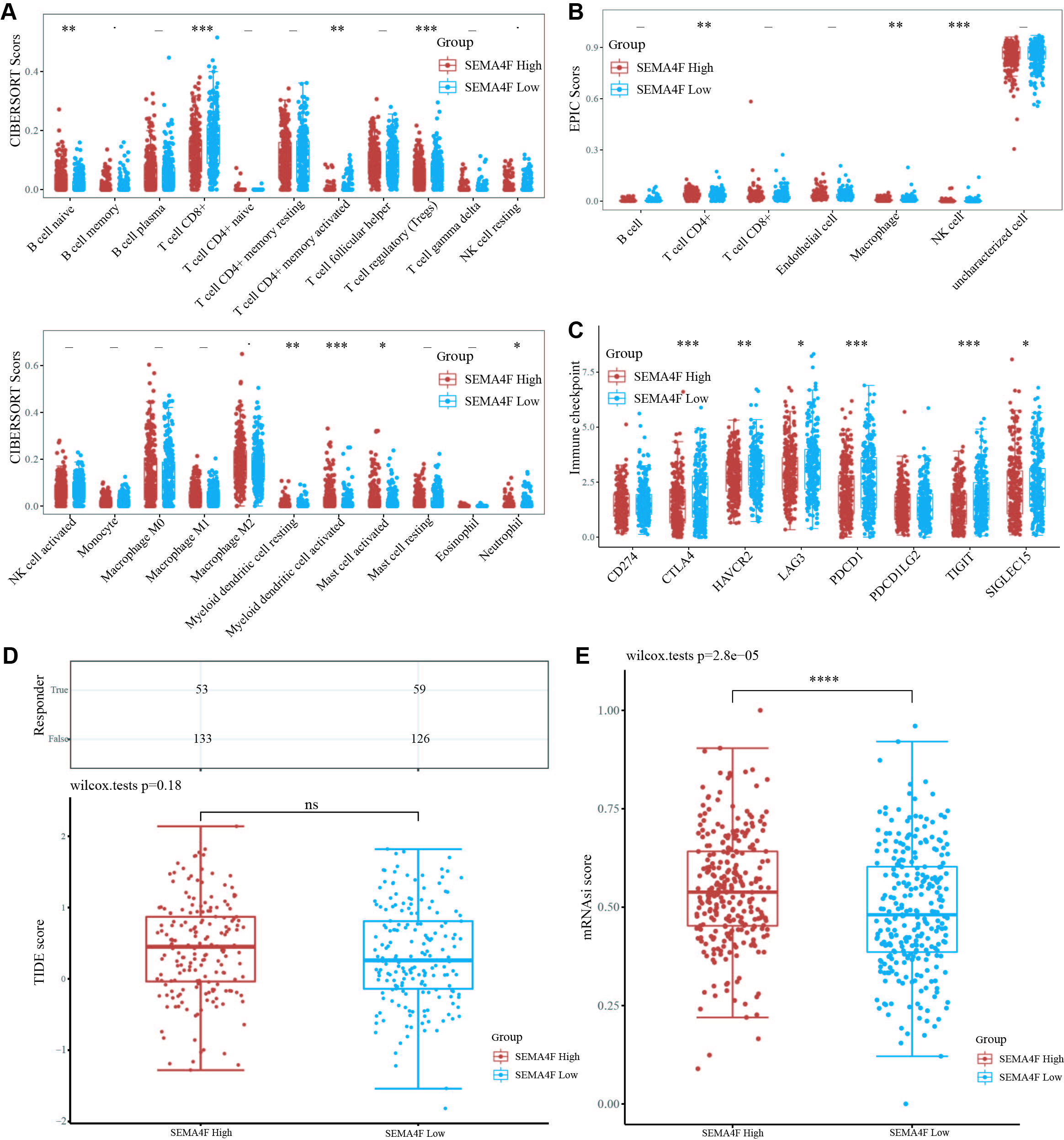

Supplement: Supplementary Figure 9 — Analysis of immune infiltration, immunological response, and stemness in two groups with high and low SEMA4F expression. [file Image_9.jpg]
